# Patients With Drug-Naive Bipolar Disorder in Remission After 8 Weeks of Treatment Had Decreased Serum Uric Acid Concentrations

**DOI:** 10.3389/fpsyt.2019.00767

**Published:** 2019-10-31

**Authors:** Jing-Xu Chen, Li-Gang Zhang, Ke-Zhi Liu, Hong-Mei Chen, Shuang-Jiang Zhou, Ning Wang, Yun-Long Tan, Shao-Li Wang, Alison Jones, Fu-De Yang, Xu-Feng Huang

**Affiliations:** ^1^Beijing Hui-Long-Guan Hospital, Peking University, Beijing, China; ^2^Department of Psychiatry, The Affiliated Hospital of Southwest Medical University, Luzhou, China; ^3^Illawarra Health and Medical Research Institute and School of Medicine, University of Wollongong, Wollongong, NSW, Australia

**Keywords:** bipolar disorder, hyperuricemia, mania, therapeutic efficacy, uric acid

## Abstract

**Background:** Evidence indicates that the serum concentration of uric acid (UA) in patients may relate both to the pathophysiology and therapeutics of bipolar disorder (BPD). The purpose of this study was to examine the changes and clinical significance of serum UA concentrations in first-episode manic patients suffering from BPD.

**Methods:** Seventy-six drug-naive patients with first-episode bipolar mania and 76 age- and gender-matched healthy subjects were recruited. Young Mania Rating Scale and Hamilton Depression Rating Scale were used to assess clinical symptoms. We tested serum UA concentrations by sandwich enzyme-linked immunosorbent assay at baseline and at the end of 8-week treatment in BPD patients and in the control group.

**Results:** After 8-week quetiapine and sodium valproate treatment, this study revealed that the serum UA concentrations in remitted patients were significantly lower than nonremitted patients; however, those remitted patients still had higher serum UA than healthy controls. We discovered that the baseline UA concentration was higher in nonremitted than remitted patients after 8 weeks of treatment. Finally, a positive association was found between serum UA and symptom relief in the first episode of manic disorder patients.

**Conclusion:** Patients with first-episode BPD had high levels of serum UA, which responds to treatment mainly in remitted patients. Our results suggest that serum UA concentrations might present potentially a trait marker in bipolar patients.

## Introduction

Bipolar disorder (BPD) is a severe and chronic mental disorder with a lifetime prevalence of approximately 1% to 2% in the general population (1). The disease is associated with a potentially devastating impact on individual well-being including social, occupational, and general functioning (2–4). Drugs such as mood stabilizers, atypical antipsychotics, and nondrug therapy, including electroconvulsive therapy, transcranial magnetic stimulation, and psychotherapy, are shown to have limited efficacy; the outcomes in a large number of patients are unsatisfactory (5–7). Therefore, the therapeutics of BPD remains an important challenge in psychiatry. This may be due to the underlying neurobiology of the diseases being still largely unclear. Improved understanding of the pathophysiological basis of BPD is required for the development of more targeted therapeutics.

Uric acid (UA) and purine regulate a number of brain activities such as cognition, memory, and mood and highly exist in the limbic regions and basal ganglia of brain (8). These brain regions have been reported to have some abnormalities in BPD through neuroimaging studies (9, 10). Growing evidence from genetic and clinical studies suggested the purinergic system might be a contributing factor in the pathophysiology of BPD (8). The purinergic system includes signaling pathways of the neurotransmitter adenosine triphosphate and the neuromodulator adenosine. Agonists of adenosine are considered to have sedative, anticonvulsant, antiaggressive, and antipsychotic properties, whereas its antagonists, for example, caffeine, may cause irritability and disrupt the sleep–wake cycle, which is a common precipitant for manic relapse (11). In addition, adenosinergic dysfunction is found in euthymic patients with BPD, and there is a significant association between higher functioning impairment and lower concentrations of adenosine in blood (12).

UA is a purine metabolite produced by the xanthine oxidoreductase from xanthine or hypoxanthine. Peripheral and central UA concentrations are highly correlated (13). An increased serum UA concentration could be due to the result of amplified purinergic turnover or reduced adenosine transmission (14, 15). Over the past decades, several studies have shown that BPD patients in all phases (manic/hypomanic, mixed depressed, or euthymic) could have an increased serum UA concentration, as compared with healthy or other mental disorder subjects (16–20). These studies have indicated altered regulation of purinergic system in BPD (21). However, no significant differences in serum UA levels were also reported between euthymic BPD and healthy subjects (12, 20, 22, 23). Also, no significant differences were found in serum UA between the patients with manic episodes and euthymic status (20, 24, 25). Therefore, whether an elevated UA concentration might be a trait marker for BPD remains to be determined.

A positive correlation was reported between serum UA levels and symptom severity in mania patients (18, 19, 26). In line with their study, a combinational therapy of valproate together with allopurinol (hyperuricemia-lowering drug) results in significant improvements in patients suffering from acute mania (27, 28). In contrast, no improvement was also reported by using allopurinol in treating manic symptoms (29, 30). Also, similar UA levels between manic state and remitted state were reported (20, 24).

In consideration of controversial data from literature, we carried out a cohort study on first-episode and drug-naive manic patients and investigated whether (1) serum UA concentrations differ among manic episode, remission after treatment, and healthy subjects, matched for age and gender; (2) treatment with an atypical antipsychotic drug (quetiapine) and mood stabilizer (sodium valproate) affects the serum UA concentrations in manic patients; and (3) there is any relationship between serum UA concentration and the therapeutic efficacy.

## Methods

### Subjects

Seventy-six inpatients were recruited from inpatients suffering BPD. The study was conducted from January 2015 to December 2017 in Beijing Hui-Long-Guan Hospital, affiliated with Clinical Medical College of Peking University, China. The inclusion criteria were subjects between 18 and 45 years old, diagnosis of a manic/hypomanic episode by the consensus of two independent senior psychiatrists according to the *Diagnostic and Statistical Manual of Mental Disorders, Fourth Edition* criteria (31), and first-episode and never-treated history, which was confirmed by collateral histories from family members. All subjects were Han Chinese. The patients with a history of affective disorder and other Axis I diagnoses such as schizophrenia and neurological illness were excluded from this study.

The control group had 76 healthy subjects and was recruited from the local community, matched with the patients for age and gender. Healthy subjects were 18 to 45 years of age without any record of personal or family history of psychiatric disorders and were Han Chinese. All participants were without any record of organic brain diseases, cardiovascular disease, chronic inflammatory disease, diabetes, nephropathy, and gout or other diseases associated with changes in serum UA concentrations, as determined by a complete medical history, physical examination, and laboratory tests. Neither bipolar patients nor control subjects were taking drugs or medications, none had alcohol abuse/dependence, and none were pregnant/breastfeeding, which could influence UA concentrations.

All subjects gave signed and informed consent that was approved by the institutional review board of the Beijing Hui-Long-Guan Hospital, Peking University.

All patients received the combination quetiapine (606.58 ± 150.85 mg, daily) plus sodium valproate (1,077.97 ± 338.77 mg, daily) treatment for 8 weeks, which was in agreement with the first-line treatment of mania in international evidence-based guidelines (32).

### Assessment

Psychopathological symptoms of patients were assessed on the day of blood sampling by four psychiatrists who were blind to the clinical status and treatment conditions. Young Mania Rating Scale (YMRS) (33) was used to measure the severity of manic symptoms before and after treatment. YMRS and the 17-item version of the Hamilton Depression Rating Scale (HDRS) (34) were used to confirm the remission during the recovery period. The patients in remission were defined by both YMRS and HDRS scores being ≤7. To ensure consistency and reliability of ratings, before undertaking the study, four psychiatrists who worked over 5 years in clinical practice simultaneously received a training session on how to use YMRS and HDRS, ensuring a correlation coefficient maintained greater than 0.8 by repeated assessments across the study.

The blood samples from all subjects were taken between 6 and 7 after overnight fasting as baseline and were taken again after 8 weeks of treatment between 6 and 7. Serum UA concentrations were assessed using sandwich enzyme-linked immunosorbent assay, which was by a commercially available kit (DRG kit cat. no. EIA-2925, Germany). The parameters potentially affecting serum UA concentrations such as metabolic parameters (triglycerides, high-density lipoprotein cholesterol, and fasting blood glucose) and kidney function (creatinine, urea) were also measured. The height, body weight, and blood pressure of all subjects were measured. Body mass index (BMI) was calculated as body weight in kilograms divided by height in meters squared. Hyperuricemia was defined as serum level of UA >420 µmol/l (7.0 mg/dl) for men and >360 µmol/l (6.0 mg/dl) for women (17). Current smoking referred to smoking at least one cigarette per day over the last 1 month before enrollment.

### Statistical Analysis

The differences of demographic data between patients and controls were analyzed by χ^2^ test or independent-samples *t* test, wherever it is appropriate. The paired *t* test was used to compare serum UA concentrations, YMRS scores, and HDRS scores between the baseline and posttreatment in the patient group. The paired *t* test was also used to compare serum UA levels in drug-naive manic patients and patients in remission after treatments. Pearson correlation coefficient was used to evaluate the relationship between serum UA levels and YMRS scores when data were in normal distribution. Independent-samples *t* test was applied to single-time-point data. When significant differences were detected, covariant analysis controlling for BMI, age, and sex and Bonferroni *post hoc* analyses for multiple comparisons were applied. Results are expressed as mean ± SD. All statistical tests were two-tailed, and *p* < 0.05 was considered as statistically significant. Statistical analyses were performed using SPSS (version 17.0).

## Results

Demographic and clinical characteristics of the study population are shown in **Table 1**. Patients and controls were matched with respect to age and gender. There were no significant differences in demographic and clinical characteristics (**Table 1**). Sixty-eight cases were mania (39 men and 29 women), and the other eight cases were hypomania (five men and three women). No differences in the levels of serum UA between mania and hypomania patients (379.74 ± 99.25 vs 368.50 ± 103.70 µmol/l, *t* = 0.34, *p* = 0.74).

**Table 1:** Demographic and clinical characteristics of drug-naive patients with first-episode manic patients and healthy controls.

Variable	Manic patients (n = 76)	Healthy controls (n = 76)	χ^2^/*t*	*p*
Age (y)	26.34 ± 8.16	26.95 ± 6.67	0.45	0.65
Gender, male, n (%)	43 (56.6)	43 (56.6)	0.00	1.00
Education (y)	12.14 ± 3.33	12.74 ± 3.40	1.08	0.28
Marital status, married, n (%)	32 (42.1)	28 (36.8)	0.44	0.51
Duration of illness (d)	37.26 ± 52.43	—		
Current smoking, n (%)	16 (21.1%)	9 (11.8%)	2.35	0.13
BMI kg/m^2^	22.14 ± 2.54	21.97 ± 2.38	0.44	0.66
Systolic BP (mm Hg)	109.34 ± 11.98	107.83 ± 9.77	0.85	0.40
Diastolic BP (mm Hg)	73.22 ± 7.99	73.16 ± 8.24	0.05	0.96
HDL-C (mmol/l)	1.32 ± 0.36	1.33 ± 0.46	0.08	0.94
TG (mmol/l)	1.24 ± 0.87	1.28 ± 0.65	0.33	0.74
FBG (mmol/l)	4.71 ± 0.67	4.68 ± 0.58	0.30	0.76
Urea (mmol/l)	4.56 ± 1.31	4.24 ± 0.96	1.76	0.08
Creatinine (µmol/l)	74.62 ± 18.61	70.04 ± 15.86	1.63	0.11
UA (µmol/l)	378.55 ± 99.08	293.39 ± 71.40	6.22	< 0.01

BMI, body mass index; BP, blood pressure; HDL-C, high-density lipoprotein cholesterol; TG, triglyceride; FBG, fasting blood glucose levels.

**Figure 1:**
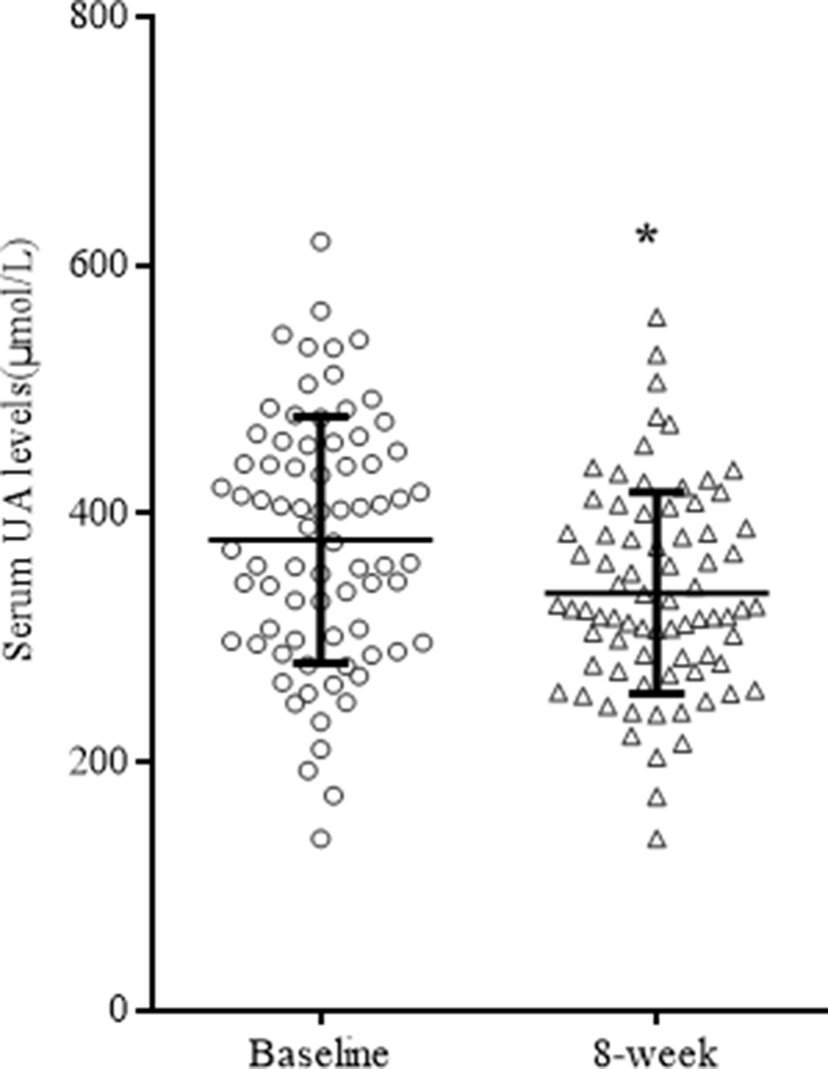
Serum UA concentarion from baseline over 8 weeks of treatment. *p < 0.05.

**Figure 2:**
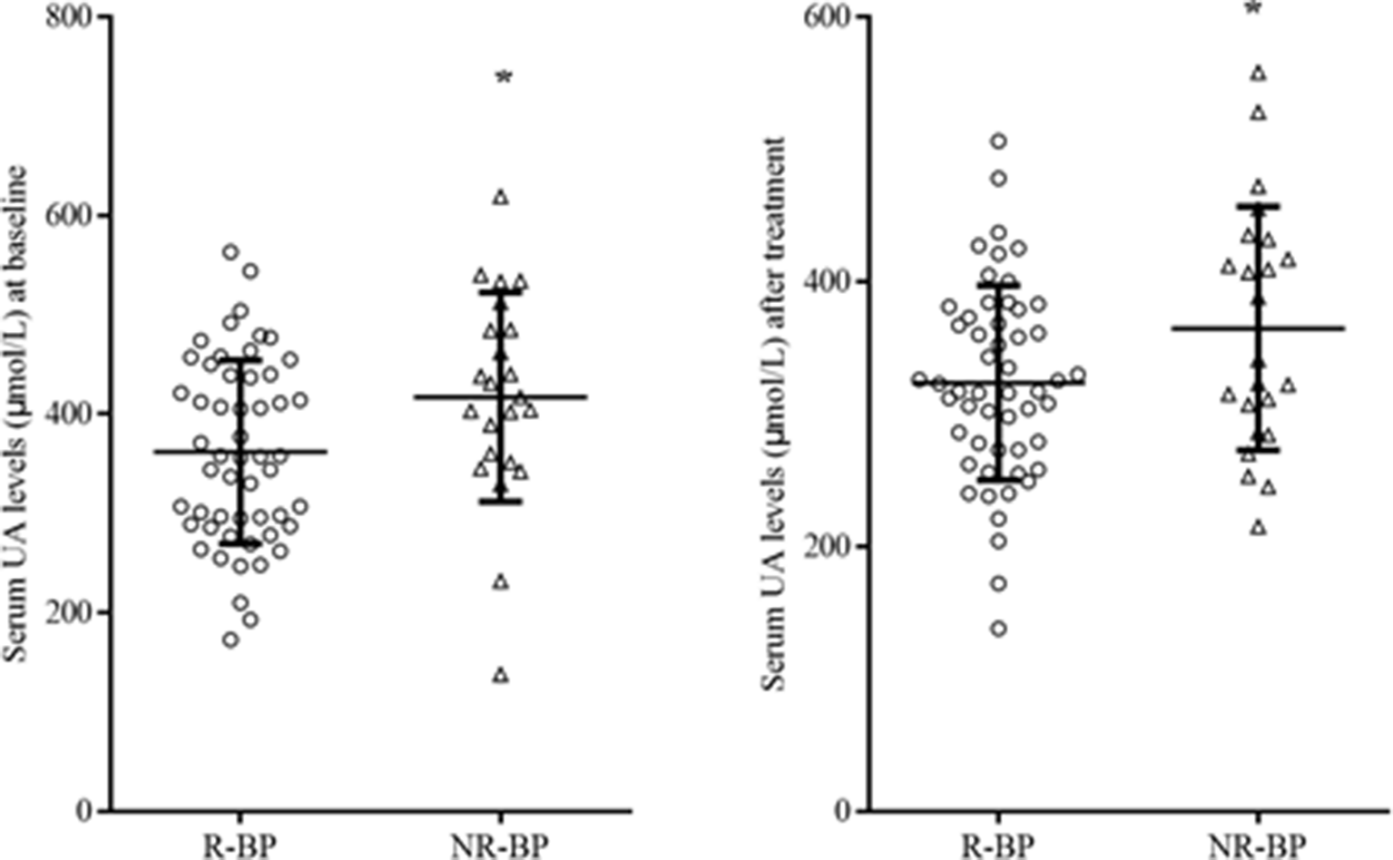
Serum UA levels R-BP (n = 53) and NR-BP (n = 23) at baseline (left panel) and after treatment (right panel). R-BP, remitted bipolar patients; NR-BP, non-remitted bipolar patients. *p < 0.05.

After 8 weeks of quetiapine and sodium valproate treatment, YMRS scores (*t* = 25.76 *p* < 0.01) and serum UA concentrations (*t* = 3.36, *p* = 0.01) were lower compared to baselines (**Table 2**, **Figure 1**). Fifty-three patients reached remission status according to the scores of YMRS and HDRS. We detected that the serum UA concentrations were significantly lower in the remission group compared to nonremission group both at baseline (361.89 ± 92.36 vs 416.96 ± 105.31 µmol/l, *t* = 2.29, *p* = 0.03) and after 8 weeks of treatment (323.57 ± 73.53 vs 364.57 ± 91.98 µmol/l, *t* = 2.07, *p* = 0.04) (**Figure 2**). These differences remained after controlling for age, gender, and BMI (all *p* < 0.05).

**Table 2:** Serum UA levels, YMRS scores, and HDRS scores among bipolar patients receiving treatment for 8 weeks.

Variable	Baseline	Week 8	*t*	*p*
UA (µmol/l)	378.55 ± 99.08	335.97 ± 81.18	3.361	0.01
YMRS scores	27.47 ± 5.23	6.44 ± 5.73	25.76	<0.01
HDRS scores	3.11 ± 3.04	4.01 ± 4.14	−1.615	0.11

UA, uric acid; YMRS, Young Mania Rating Scale; HDRS, Hamilton Depression Rating Scale.

**Figure 3:**
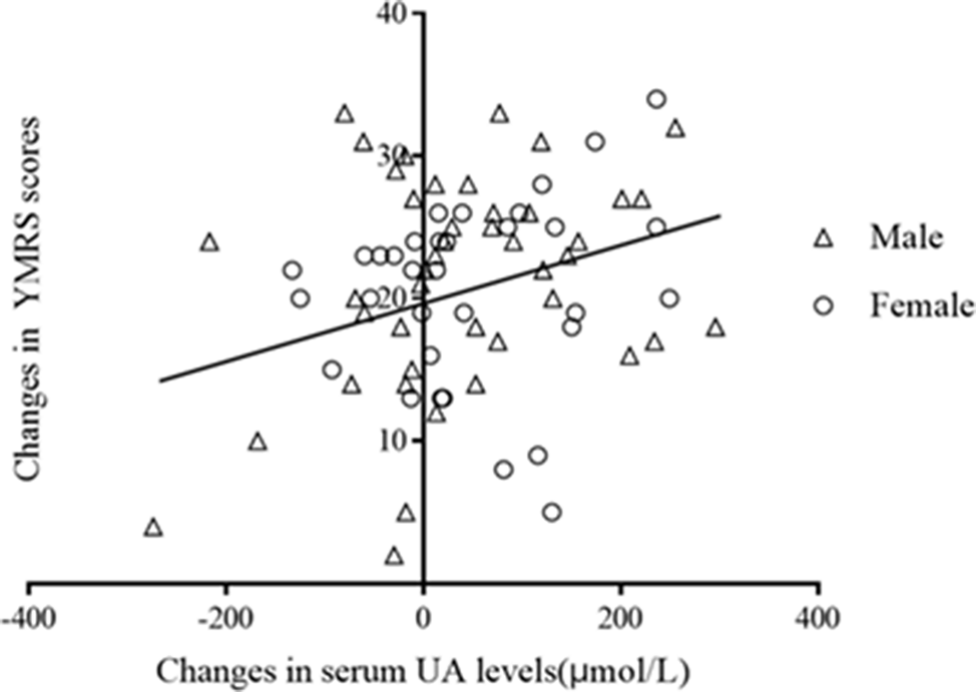
Correlation between changes in serum UA levels and that in YMRS scors after treatment (r = 0.25, *p* = 0.03).

Pearson correlation analysis showed that serum UA concentrations were not correlated with severity of symptoms, which were assessed by YMRS at both baseline (*r* = 0.18, *p* = 0.12) and at the end of 8 weeks of treatment (*r* = 0.21, *p* = 0.07). However, there was a significant correlation between changes in serum UA concentrations and that of the change of the YMRS scores after treatments (pretreatment − posttreatment, *r* = 0.25, *p* = 0.03; **Figure 3**). The correlations remained significant after controlling for the baseline YMRS score and UA levels (*r* = 0.23, *p* = 0.047).

**Figure 4:**
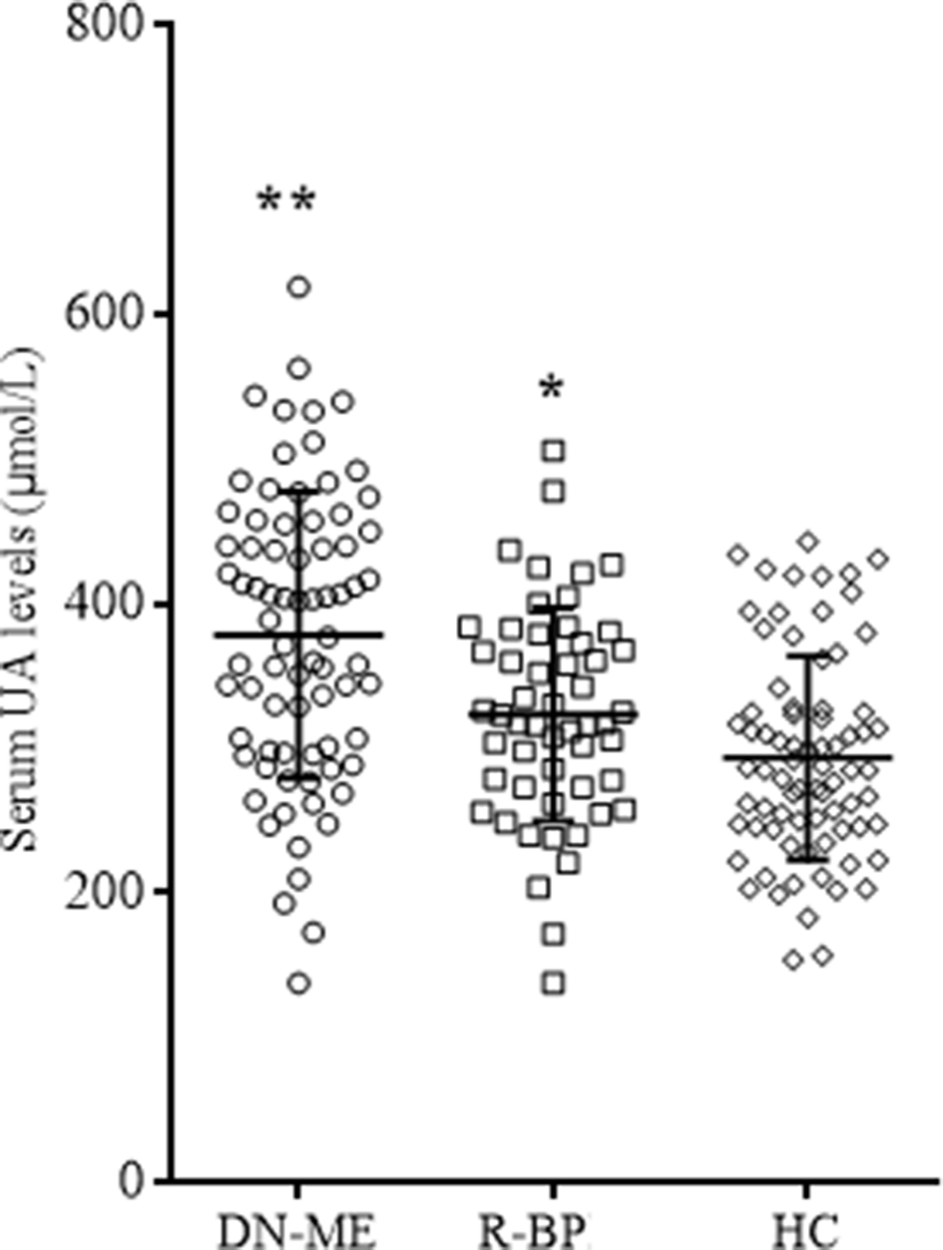
Serum UA levels in different groups. FE-MP, fist-episode manic patients (n = 76); R-BP, remitted bipolar disorder (n = 53); HC, healty controls (n = 76). *p < 0.05 comparing with HC; **p < 0.01 comparing with HC.

The prevalence rate of hyperuricemia between the remitted patients and healthy controls did not differ (17.0% and 10.5%, χ^2^ = 1.14, *p* = 0.29). The prevalence rate of hyperuricemia in all patients at baseline was 40.9%, which was significantly higher than the remitted patients and healthy controls (χ^2^ = 18.25, *p* < 0.01; and χ^2^ = 8.27, *p* < 0.01, respectively). A paired-sample *t* test revealed that serum UA levels of the remitted patients were significantly higher than these patients in drug-naive phase (**Tables 1** and **2**, *t* = 2.71, *p* < 0.01). Independent-samples *t* test showed that serum UA levels in healthy controls were lower than first-episode drug-naive patients (**Table 1**, *t* = 6.22, *p* < 0.01) and the remitted patients after 8-week treatments (*t* = 2.49, *p* = 0.01; **Figure 4**). Covariance analysis showed that the serum UA differences between healthy controls and manic patients and remitted patients remained statistically significant after adjusting for gender, age, and BMI (all *p* < 0.01; **Table 1** and **Figure 4**).

## Discussion

This study analyzed the relationship of system levels of UA to measures of YMRS and HDRS in first-episode manic patients suffering from BPD prior and after 8-week quetiapine and sodium valproate treatment. Our results revealed that the serum UA levels in remitted patients were significantly lower than nonremitted patients, although they were still had higher than healthy controls. Furthermore, we showed that the baseline UA was significantly higher in nonremitted than remitted patients after 8 weeks of treatment. Also, a positive association was found between serum UA and symptom relief in these patients.

In the past few years, a number of studies have investigated the changes of UA in patients suffering BPDs; however, the data are inconsistent and even controversial (20, 22, 35, 36). This may be due to various confounders involved in studies because the nature of BPD is a complex, chronic, and heterogeneous psychiatric disease (37). Two studies have investigated serum UA levels in first-episode manic patients with the sample sizes of 20 based on the criteria of *Diagnostic and Statistical Manual of Mental Disorders, Fourth Edition* and 31 based on the criteria of *International Classification of Diseases, 10th Revision* (26, 38). Both studies have had relatively small sample size without taking into consideration of confounders such as the levels of creatinine, cholesterol, and glucose and BMI, which could have influenced UA levels (39, 40). We conducted a prospective study on a cohort of 68 first-episode manic patients controlling for potential confounders including metabolic and kidney function–related variables. From this study, we suggest that a possible contributing factor could be due to discrepancy of baseline levels of UA in the BPD patient cohort studied.

Overall, recent studies support that the patients suffering BPD at manic phase have significantly higher levels of serum UA during both acute and chronic phases compared with healthy controls (24, 28, 38, 41). Also, the serum UA levels were higher in mania phase than depression and remission phases in the same patients suffering from BPD (19, 25, 42). Consistent with these results, our study showed that first-episode drug-naive patients in manic phase had significantly higher serum UA than those of patients in remission and healthy controls. Therefore, we support that increased UA levels were associated with a state of manic episode, which may be used as a routine check in the manic phase of BPD (13).

Psychotropic and antipsychotic drugs could have different effects on serum UA levels. Lithium and carbamazepine reduce UA serum levels (43, 44). Zotepine reduces UA levels more than risperidone and haloperidol (45, 46). Conversely, it has been reported that olanzapine increases UA levels in moderate mania (47, 48). The present study observed that bipolar patients in mania phase treated with quetiapine and valproate decreased serum UA, which was in consistence with a report of a previous study (18). Previous study showed that adjunctive allopurinol reduces UA and increases remission rate in manic patients (49). Similarly, we found that decreased UA was in parallel with an improvement of YMRS scores in mania patients. It was possible that UA may have contributed to the severity of mania symptoms, while reduced UA helped relief of manic symptoms. One explainable mechanism was that alleviated oxidative stress by lowering UA may have led to the effect of antimanic drug because an elevated UA is known to induce oxidative stress, which may worsen the pathophysiology of manic disorder (50–53).

The present study was the first to investigate the serum UA levels in patients suffering from the first manic episode who have achieved remission after 8 weeks of treatment. We found that serum UA levels in remitted patients were higher than those in healthy controls after adjusting for age, gender, and BMI, which supported the idea that altered purinergic regulation may be involved in the genesis of BPD (19, 21, 24, 54). In addition, several studies have reported higher levels of UA in the depressive episodes of BPD compared with healthy controls (19, 24, 54). Therefore, an increased UA may be a predisposition in bipolar patients as a trait marker, which is independent of some current or past affective episodes. On the other hand, it was noted that some euthymic patents had similar UA levels as healthy controls particularly if the patients had a longer duration of illness, high number of affected mood episodes, and longer mood stabilizer exposure (12, 20, 22, 23).

The present study had several limitations. First, our data should be seen as a pilot study instead of generalized clinical evidence or relevance because our study had relatively small sample size, especially of female patients from one district of China. Second, bipolar patients with depressive episodes or recurrent episodes (possibly with years of pharmacological intervention) were not enrolled in this study. Third, the prevalence of cigarette smoking was normally higher in patients with BPD than healthy controls, but how smoking affects serum UA in BPD patients is still largely unknown (55, 56). Fourth, all inpatients had the same dietary profile; however, healthy subjects might have a different dietary profile, which could affect the comparison of serum UA between patients and healthy controls (57). Lastly, only one time point in remission was checked; thus, the time course of UA response to therapy remains unknown.

## Conclusions

Bipolar patients at the first-episode drug-naive phase had significantly higher levels of serum UA. Patients in remission after 8 weeks of treatment had significantly reduced serum UA, while it was still higher than healthy controls. Patients in nonremission after 8 weeks of treatment had significantly less reduction in serum UA compared with those of remitted patients. Therefore, high levels of serum UA were a pathological condition of bipolar disease at manic phase, contributed to the severity of symptoms, and were reduced in remitted patients, but not in nonremitted patients. Furthermore, serum UA levels might be potentially a trait marker in bipolar patients.

## Data Availability Statement

The datasets used and/or analyzed during the current study are available from the first author on reasonable request.

## Ethics Statement

This research was approved by the Human Ethics Committee of Beijing Hui-Long-Guan Hospital, China. All patients were provided with written informed consent. Participation was voluntary and participants could withdraw at any time from the study.

## Author Contributions

Conception of the study: J-XC, F-DY, and X-FH were involved in study design. J-XC, L-GZ, K-ZL, H-MC, and S-JZ were responsible for recruiting the patients, performing the clinical rating, collecting all samples, and helping with statistical analysis and manuscript preparation. X-FH, J-XC, AJ, Y-LT, and F-DY were involved in interpreting the data, intellectual input, and writing and editing the paper. All authors have read and approved the manuscript.

## Funding

This study was supported by the Capital Foundation of Medicine Research and Development (2018–3–2132); the Special Foundation of Beijing Municipal Science & 258 Technology Commission, China (Z131107002213099); and Scientific Research Foundation of Sichuan Province (2017JY0322).

## Conflict of Interest

The authors declare that the research was conducted in the absence of any commercial or financial relationships that could be construed as a potential conflict of interest.

## Abbreviations

BMI, body mass index; BP, blood pressure; BPD, bipolar disorder; CI, confidence interval; DSM-IV, Diagnostic and Statistical Manual of Mental Disorders, 4th Edition; FBG, fasting blood glucose; HDL-C, high density lipoprotein cholesterol; OR, odds ratio; SPSS, Statistical Package for the Social Sciences; UA, uric acid.
